# Case report: Spinal drop metastasis of IDH-mutant, 1p/19q-codeleted oligodendroglioma

**DOI:** 10.3389/fneur.2022.1086591

**Published:** 2022-12-16

**Authors:** Ahmet Kursat Karaman, Bora Korkmazer, Nil Urganci, Gülçin Baş, Serdar Arslan, Nil Comunoglu, Mehmet Murat Hanci, Osman Kızılkılıç

**Affiliations:** ^1^Department of Radiology, Sureyyapasa Chest Diseases and Thoracic Surgery Training Hospital, Istanbul, Turkey; ^2^Division of Neuroradiology, Department of Radiology, Istanbul University-Cerrahpasa, Istanbul, Turkey; ^3^Department of Pathology, Cerrahpasa Medical Faculty, Istanbul University-Cerrahpasa, Istanbul, Turkey; ^4^Department of Neurosurgery, Cerrahpasa Medical Faculty, Istanbul University-Cerrahpasa, Istanbul, Turkey

**Keywords:** oligodendroglioma, IDH1 (R132H) mutation, drop metastases, spinal metastasis, 1p/19q-codeletion

## Abstract

**Background:**

Symptomatic spinal metastases of oligodendroglioma are rare. Moreover, none of the previously published cases demonstrated the typical IDH mutation and 1p/19q-codeletion for this glial tumor. This case presents an IDH mutant, 1p/19q-codeleted oligodendroglioma with multiple spinal drop metastases.

**Case description:**

We report a case of a 55-year-old woman with left frontal grade 3 oligodendroglioma diagnosed 3 years ago. No tumor recurrence was observed in post-operative follow-up MRI examinations. However, she was admitted to our institution again with severe low back pain. Gadolinium enhanced MRI of the spine revealed an intradural, extramedullary metastatic lesion between T11–L1 levels and multiple enhancing metastatic tumor deposits around cauda equine roots between L4–S1. T11–T12 midline laminectomy was performed and gross total resection of metastatic lesions was achieved. Final histological diagnosis of the spinal lesions was WHO Grade 3 Oligodendroglioma, IDH-mutant, 1p/19q-codeleted.

**Conclusion:**

This case is the first molecularly-defined spinal metastatic oligodendroglioma. The possibility of drop metastasis should be kept in mind in oligodendroglioma patients with spinal cord-related symptoms. There is no standard approach for the diagnosis and treatment of spinal metastases of this type of glial tumor.

## Introduction

Oligodendrogliomas are rare neuroepithelial tumors accounting for 2–5% of all brain tumors in adults (1). In the 5th edition of the WHO classification of CNS tumors, they are defined as diffuse gliomas that demonstrate IDH mutation and 1p/19q-codeletion. The characteristic 1p19q deletion in these tumors is also associated with greater response

to treatment and better clinical outcome (2). Microscopic spread of oligodendrogliomas *via* CSF to spinal subarachnoid spaces is a well-defined and relatively common event, reported up to 14%. However, clinically significant macroscopic spinal cord metastases are rare. In addition, to the best of our knowledge, 1p19q deletion has not been described in previously reported cases of oligodendroglioma with spinal drop metastasis (3, 4).

In this article, we report a case of IDH-mutant, 1p/19q-codeleted oligodendroglioma presenting with multiple spinal drop metastases 3 years after initial diagnosis.

## Case presentation

**Figure 1:**
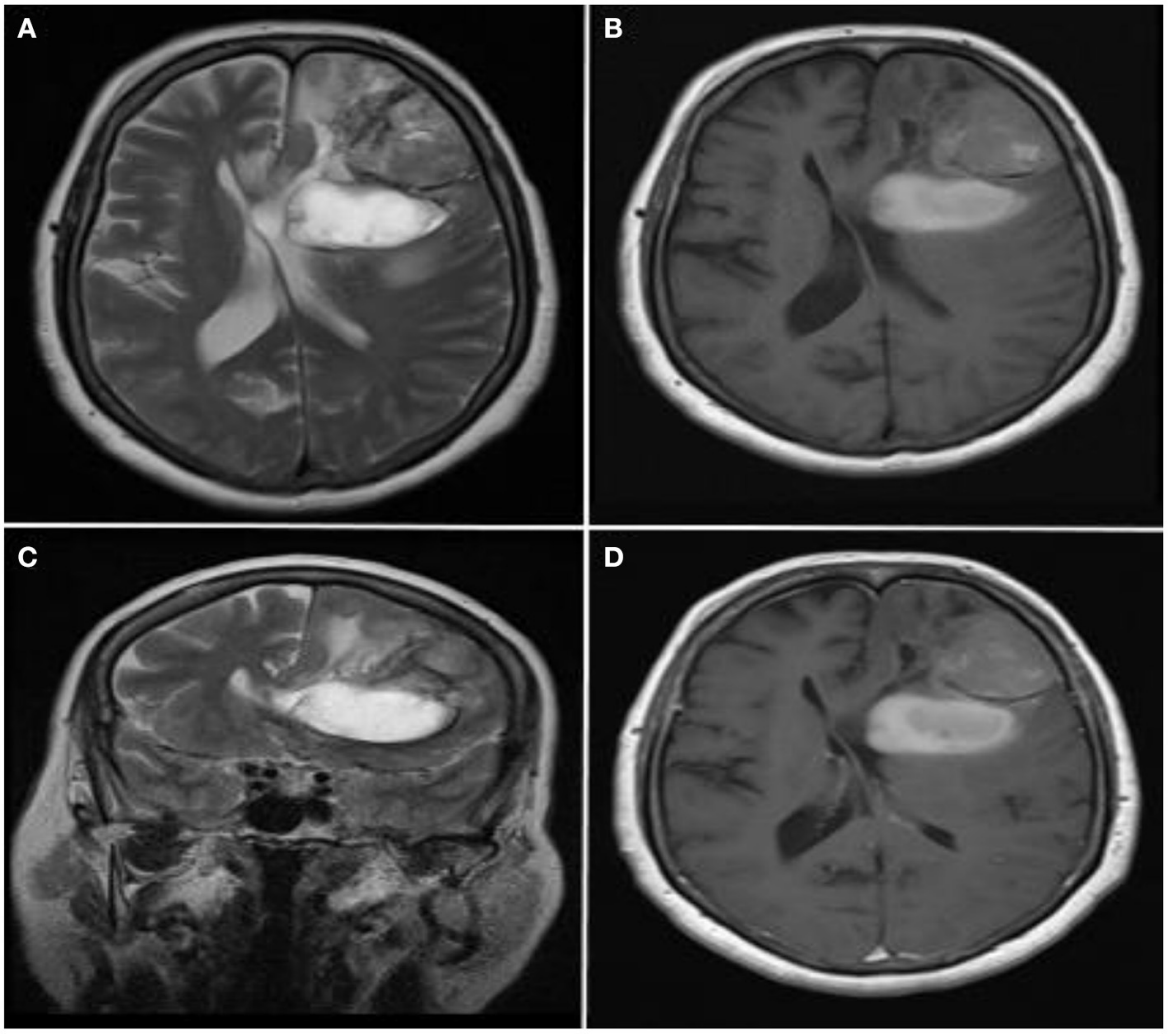
Radiological images of the primary lesion. **(A–C)** Axial T2-weighted, axial T1-weighted and coronal T2-weighted MR images show a large, heterogeneous, left lateral ventricle compressing mass and mild perilesional edema in the left frontal lobe. Hemorrhagic appearance is observed in the posterior-inferior part of the mass (hyperintense signal on T1-weighted and T2-weighted images). **(D)** There was no enhancement in the mass on the Gd-enhanced T1-weighted MR image.

A 55-year-old woman presented with a headache that had worsened over the past few months in June 2019. MRI of the brain revealed a large heterogeneous mass of 6.5 × 5.5 × 4.2 cm in the left frontal lobe and mild perilesional edema. The mass was compressing the left lateral ventricle and there was hyperintense signal on T1W and T2W images consistent with hemorrhage in its part in this region. No contrast enhancement was observed in the mass on post-contrast MR images (Figure 1). Total re-section of the tumor was performed and histopathological diagnosis was oligodendroglioma, WHO grade 3, IDH-mutant, and 1p/19q-codeleted. The patient was asymptomatic in the postoperative period. Adjuvant treatment (cranial radiotherapy combined with temozolomide) was recommended by oncologists, but she refused. No tumor recurrence was observed in follow-up MRI examinations.

**Figure 2:**
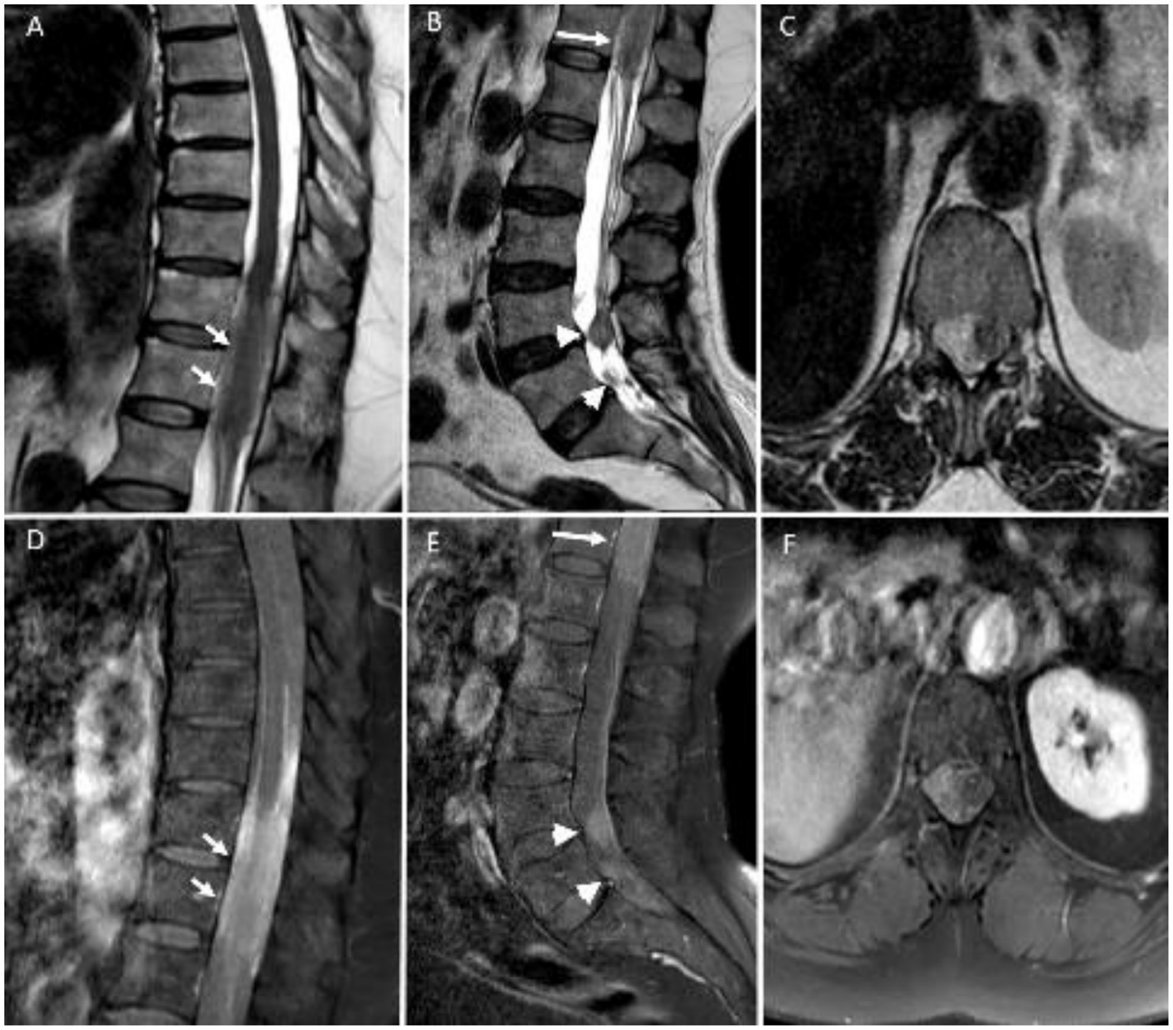
Sagittal T2-weighted **(A,B)** and post-contrast T1-weighted **(D,E)** MR images demonstrate multiple metastatic lesions with contrast enhancement between T11–L1 levels (long arrows) and around cauda equina roots (arrowheads). Axial T2-weighted **(C)** and post-contrast T1-weighted **(F)** images at T12 level show an extramedullary metastatic lesion compressing the spinal cord.

However, in July 2022 (36 months after initial diagnosis), the patient was admitted to our institution with a 3-week history of severe low back pain. Neurological examination demonstrated mild paraparesis. No sensory deficit was found and deep tendon reflexes were normal. Gadolinium enhanced MRI of the spine revealed an intradural extramedullary lesion compressing the spinal cord between T11–L1 levels and multiple enhancing tumor deposits attached to cauda equina roots between L4–S1 levels (Figures 2A–F). There was no evidence of metastases outside the central nervous system. T11–T12 midline laminectomy was performed to decompress the spinal cord and obtain histological samples. A gray-hemorrhagic, soft tumoral extra medullary lesion was observed following the opening of the dura. Also, tumoral nodules surrounding the cauda equina roots were observed. Subtotal resection of all visible tumor was achieved. Early postoperative course was uneventful. Spinal radiotherapy was planned for residual tumor between T11–L1 levels and tumor deposits between L4–S1 levels.

**Figure 3:**
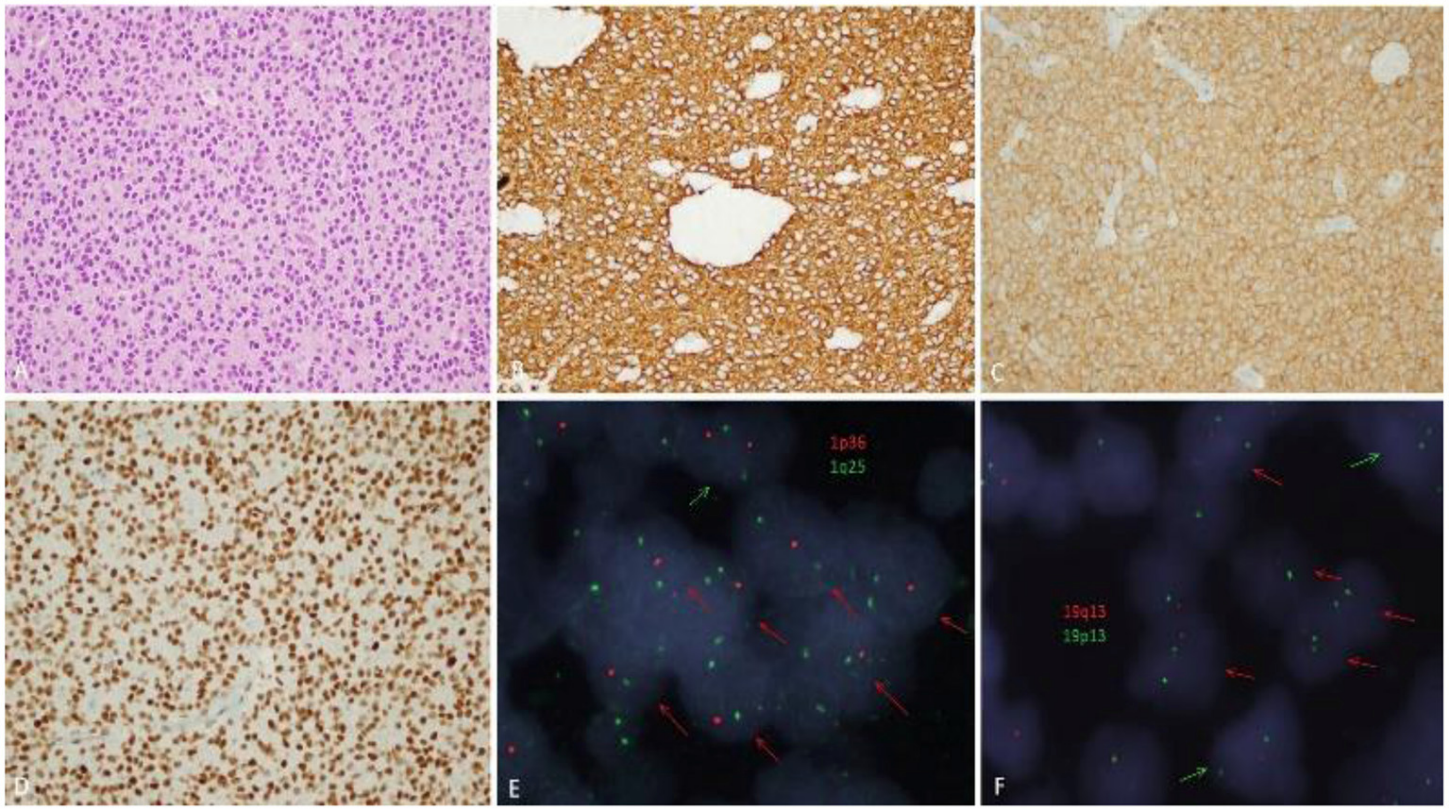
Pathological findings. **(A)** Microscopic examination (H & E, ×400) showed small-medium sized tumor cells with round nuclei and perinuclear haloes. **(B)** GFAP was diffusely positive in tumor cells. **(C)** Immunohistochemistry for IDH-1 R132H was positive (mutant). **(D)** ATRX immunohistochemistry showed retained wild-type expression. **(E,F)** 1p19q-codeletion in oligodendroglioma *via* fluorescence *in situ* hybridization. **(E)** 1p deletion of oligodendroglioma tumor cell nuclei (two green signals for 1q25, one red signal for 1p36). **(F)** 19q deletion of oligodendroglioma tumor cell nuclei (two green signals for 19p13 and one red signal for 19q13). Red arrows showed cells with deletion, and green arrows showed cells without deletion.

Histopathological examination revealed that the tumor consisted of small-medium cells with small, rounded nuclei, perinuclear clearing, and the presence of increased cellularity, pleomorphism, brisk mitosis and focal necrosis. Vascular endothelial proliferation was absent (Figure 3A). Immunohistochemical staining showed positive results for GFAP, EMA, Olig2 and mutant IDH-1 (Figures 3B,C). Immunohistochemistry for ATRX showed retained wild-type expression (Figure 3D). Ki-67 proliferation index was 30%. Fluorescence *in situ* hybridization studies revealed 1p/19q co-deletion (1p/1q ratio 85%, 19q/19p ratio 65%) in tumor cells (Figures 3E,F). Final histological diagnosis was WHO Grade 3 Oligodendroglioma, IDH-mutant, 1p/19q-codeleted.

## Discussion

Oligodendrogliomas are the rarest type of primary brain tumor associated with distant metastases. In these tumors, the most frequent metastatic sites are bone, bone marrow and lymph nodes (5, 6). The occurrence of spinal drop metastases is exceptional, and according to our extensive literature search, there are 18 previously described cases with confirmed pathological diagnosis in the literature. However, none of the reported cases were evaluated for 1p/19q code-deletion or IDH mutation status as defined in the WHO 2021 CNS tumor classification (Table 1) (4, 7–20). Thus, the present case, with IDH mutation and 1p/19q deletion, is the first 'true' (molecularly proven) spinal drop metastasis of oligodendroglioma.

**Table 1:** Summary of previously reported oligodendroglioma cases with spinal drop metastasis.

**Patient**	**Age/gender**	**Primary tumor site**	**Spinal metastases**	**Interval** **(from initial diagnosis)**	**Histological** **diagnosis (WHO CNS5)**	**References**
1	29/M	L lateral ventricle	Diffuse	3 years	Grade 2, OG NOS	Beck and Russell (7)
2	42/M	R frontal	Diffuse	Post-op	Grade 2, OG NOS	Beck and Russell (7)
3	6.5/F	R frontal	Diffuse	3 months	Grade 2, OG NOS	Beck and Russell (7)
4	38/M	R temporoparietal	Diffuse	6 years	Grade 3, OG NOS	Trowbridge (8)
5	30/M	R frontal	Cauda equina	5 years	Grade 3, OG NOS	Strang and Nordenstam (9)
6	43/F	L frontal	T3–T5	7 years	Grade 2, OG NOS	Reggiani (10)
7	27/F	R frontal	Diffuse	6 months	Grade 3, OG NOS	Voldby (11)
8	11/M	Third ventricle	C1–C5, ekstramedullary	2 years	Grade 2, OG NOS	Arseni et al. (12)
9	39/M	R temporal	T1–T3	15 months	Grade 2, OG NOS	Arseni et al. (12)
10	13/F	R parietal	T6–T8, intramedullary	8 months	Grade 2, OG NOS	Van Velthoven et al. (13)
11	40/M	L temporal	T11, extramedullary	6 years	Grade 2, OG NOS	Wallner et al. (14)
12	61/F	L frontal	Diffuse	20 months	Grade 3, OG NOS	Ng et al. (15)
13	49/M	R/parietal	C7–T2, intramedullary	14 months	Grade 2, OG NOS	McBryde et al. (16)
14	67/F	L lateral ventricle	Cauda equina	Synchronous	Grade 3, OG NOS	Natale et al. (17)
15	40/M	R frontal	Diffuse	1 year	Grade 3, OG NOS	Ozisik et al. (18)
16	73/F	Cerebellar	C2–C4, intramedullary	18 months	Grade 3, OG NOS	Oshiro et al. (19)
17	37/M	L. frontoparietal	T12–L1, intramedullary and nerve roots	4 years	Grade 3, OG NOS	Elefante et al. (4)
18	46/M	L frontal	T12–L1, intramedullary and nerve roots	2 years	Grade 3, OG NOS	Carrizosa et al. (20)
19	55/F	L frontal	T11–L1, extramedullary, cauda equina	3 years	Grade 3 OG, IDH-mutant, 1p/19q-codeleted	Present case

OG, oligodendroglioma; NOS, not otherwise specified.

Most spinal metastases from oligodendroglioma are extramedullary. However, intramedullary localization was observed in 5 of 18 reported cases (4, 13, 16, 19, 20). In addition, drop metastases may be diffuse, filling the entire spinal canal, or may be one or more tumor deposits involving a relatively short spinal segment (4). In our case, as in most of the previously described cases, extramedullary metastases were described and multiple tumoral lesions were detected between T11–L1 levels and around cauda equina fibers.

The primary advocated mechanism for metastasis to meningeal surfaces along the spinal cord is the transition of tumor cells to the cerebrospinal fluid (CSF) by disrupting the pia and ependyma, and then their seeding by following the normal CSF pathway with the help of gravitational forces. Previous cranial surgery and/or CSF shunting may be the initiating factor for this mechanism. Disruption of the integrity of the meningeal barriers secondary to surgical intervention may cause subarachnoid spread. Moreover, disruption of the blood-brain barrier integrity may facilitate hematogenous extracranial metastasis (21, 22). Except for the case reported by Natale et al. (4, 17), all cases in the published literature had a history of previous brain tumor surgery and spinal metastases occurred in the early or late period (3 months−7 years) after surgery. Spinal drop metastases were detected 36 months after primary tumor surgery in our case. Besides, spread along the Virchow-Robin spaces of the spinal cord is advocated for intramedullary metastases (17).

Tumor-related intrinsic factors that may play a role in subarachnoid metastasis of glial tumors are the malignant potential and location of the tumor. In general, most of the glial tumors that metastasize to the spinal cord are high-grade diffuse gliomas, as in our case (23). For oligodendrogliomas, however, the situation appears different; In half (9/18) of oligodendroglioma cases with spinal metastases, the primary tumor is low grade (WHO grade II) (4). In addition, the 1p/19q-codeletion in these tumors is not correlated with metastatic relapse. The contribution of this molecular alteration, typical for the diagnosis of oligodendroglioma, to distant metastatic potential is explained by its association with prolonged survival. As the patient's survival increases, the probability of tumor progression and distant metastasis increases (24). In our case, spinal metastasis occurred 3 years after the primary tumor diagnosis. As mentioned above, since the spread *via* CSF is the main pathway in spinal metastasis of oligodendroglioma, intraventricular location of the lesion or contact with the subarachnoid space may be a remarkable factor (4, 18). In 18% (3/18) of reported cases, the lesion location is intraventricular (7, 12, 17). Moreover, close proximity to the pericerebral subarachnoid area/ventricle was observed in most of the cases (17, 18). In our case, the primary lesion was located close to both the pericerebral subarachnoid space and the ventricle.

Since spinal drop metastasis of oligodendrogliomas is a rare entity, routine imaging of the entire neuraxis is not recommended in asymptomatic cases. In patients with spinal symptoms, imaging of the entire CNS with MRI seems reasonable (18, 22). Management of spinal metastases should be done in a case-based fashion. Surgery can be used for decompression, especially in cases without diffuse involvement. On the other hand, spinal radiotherapy can be performed in cases who are not suitable for surgical intervention (18, 19). In general, the presence of spinal metastases associated with intracranial oligodendrogliomas is considered as a poor prognostic factor. However, there is scarce data regarding the long-term follow-up results in the literature. In addition, the prognostic effect of this condition is controversial, as there is no spinal metastatic 'true' oligodendroglioma with the 1p/19q-codeletion (4, 15, 17).

## Conclusions

Spinal drop metastasis of oligodendroglioma is not common. The fact that previously reported cases were not evaluated for the typical 1p/19q-codeletion and IDH mutation for this tumor in the current WHO classification suggests that the primary lesion may be other diffuse glial tumors in some of these cases. Therefore, the exact frequency of spinal metastatic oligodendrogliomas and their relationship with clinical outcome are unknown. Furthermore, there is no standard approach for the diagnosis and treatment of spinal metastases.

## Data availability statement

The original contributions presented in the study are included in the article/supplementary material, further inquiries can be directed to the corresponding author.

## Ethics statement

Ethical review and approval was not required for the study on human participants in accordance with the local legislation and institutional requirements. The patients/participants provided their written informed consent to participate in this study. Written informed consent was obtained from the individual(s) for the publication of any potentially identifiable images or data included in this article.

## Author contributions

Conception and design: AK, BK, and OK. Data acquisition: SA, NU, and GB. Analysis and interpretation of data: BK, NU, and GB. Article drafting: AK and BK. Study supervision: OK, MH, and NC. Approved the final version of the manuscript on behalf of all authors: AK. Reviewed submitted version of the manuscript and critical revision of the article: all authors. All authors contributed to the article and approved the submitted version.
